# A New High-Efficiency Fertilization System from Waste Materials for Soil Protection: Material Engineering, Chemical-Physical Characterization, Antibacterial and Agronomic Performances

**DOI:** 10.3390/ma18153492

**Published:** 2025-07-25

**Authors:** Martina Napolitano, Gianluca Malavasi, Daniele Malferrari, Giulio Galamini, Michelina Catauro, Veronica Viola, Fabrizio Marani, Luisa Barbieri

**Affiliations:** 1 Department of Engineering “Enzo Ferrari”, University of Modena and Reggio Emilia, Via P. Vivarelli 10, 41125 Modena, Italy; luisa.barbieri@unimore.it; 2 Department of Chemical and Geological Sciences, University of Modena and Reggio Emilia, Via G. Campi 103, 41125 Modena, Italy; daniele.malferrari@unimore.it (D.M.); giulio.galamini@unimore.it (G.G.); fabrizio.marani@lb-technology.com (F.M.); 3 BIOGEST-SITEIA, Piazzale Europa 1a, 42124 Reggio Emilia, Italy; 4 Department of Engineering, University of Campania “Luigi Vanvitelli”, Via Roma 29, 81031 Aversa, Italy; michelina.catauro@unicampania.it (M.C.); veronica.viola@unicampania.it (V.V.); 5 LB Officine Meccaniche S.p.A., Via Pedemontana 166, 41042 Fiorano Modenese, Italy

**Keywords:** slow-release fertilizers, matrix-based SRFs, organic matrix, material engineering, circular economy

## Abstract

The development of slow-release fertilizers (SRFs) based on production residues is a promising strategy to improve nutrient use efficiency and promote circular economy practices in agriculture. In this study, a series of experimental formulations were designed and tested using pumice scraps, liquid and dried blood, and bone meal, aiming at producing sustainable and low-cost N-P-K SRFs. These were processed through mixing and granulation, both in the laboratory and on a semi-industrial scale. The formulations were evaluated through release tests in 2% citric acid solution simulating the acidic conditions of the rhizosphere, and in acetic acid to assess potential nutrient leaching under acid rain conditions. The results showed a progressive cumulative release of macronutrients (NPKs), ranging from approximately 8% at 24 h to 73% after 90 days for the most effective formulation (WBF6). Agronomic trials on lettuce confirmed the effectiveness of WBF6, resulting in significant biomass increases compared with both the untreated control and a conventional fertilizer. The use of livestock waste and minerals facilitated the development of a scalable product aligned with the principles of sustainable agriculture. The observed release behavior, combined with the simplicity of production, positions these formulations as a promising alternative to conventional slow-release fertilizers.

## 1. Introduction

The rapid increase in global population over the past century has led to a dramatic rise in food demand. Consequently, agricultural systems are under growing pressure to produce higher yields [1,2], resulting in increased reliance on intensive farming practices. Fertilizers, particularly those containing nitrogen (N), phosphorus (P), and potassium (K), are essential for plant growth. Their widespread application has significantly boosted crop yields, playing a crucial role during the Green Revolution in enhancing global food security [3]. The effectiveness of inorganic fertilizers, especially in promoting short-term productivity, has made them indispensable in modern agriculture. By supplementing soils with concentrated nutrients, farmers were able to maximize production. As a result, the use of mineral fertilizers spread globally, transforming agricultural landscapes and altering natural soil nutrient cycles [4,5].

Although synthetic fertilizers have significantly increased agricultural output in the short term, an over-reliance on inorganic fertilizers containing nitrogen (N) and phosphorus (P) has led to numerous negative ecological consequences. A key concern is the decline of soil health caused by high agrochemical concentrations, which alter the chemical, physical and biological properties of the soil. Overuse of these fertilizers can lead to the depletion of soil organic matter (SOM), ultimately reducing soil fertility, moisture retention and structural integrity over time [4,5,6,7,8].

Another significant effect of excessive fertilizer use is water pollution, especially from nitrogen losses via runoff and leaching. This is especially caused by inefficient nutrient utilization, with nutrient use efficiency (NUE) being estimated at only 50–60% [3,9,10,11]. These nitrogen compounds, when leached into water bodies, contribute to the eutrophication of aquatic ecosystems, causing harm to aquatic life and worsening water quality [12,13,14,15].

The effects of nitrogen overfertilization on the environment extend beyond soil and water. Because nitrous oxide (N_2_O), a powerful greenhouse gas, is released into the atmosphere during the volatilization and denitrification processes of nitrogen fertilizers, they directly contribute to greenhouse gas emissions [16,17,18,19,20].

On the other hand, it is estimated that crops consume only about 10–25% of the phosphorus added to the soil [21]. Excessive application of phosphate fertilizers not only leads to resource wastage and increased costs for farmers [22] but also accelerates the depletion of finite phosphate rock reserves, rendering phosphorus use unsustainable in the long term [23,24]. Although phosphorus has low solubility, and its discharge into water bodies is typically limited, it can still contribute to eutrophication, particularly in the presence of acidic soils that enhance its mobility [21,25,26,27,28,29,30]. Finally, the use of phosphorus fertilizers derived from phosphate rocks, which naturally contain various heavy metals, results in additional pollution effects [31,32,33].

Given the environmental concerns associated with conventional fertilizers, recent innovations in fertilizer formulations aim to enhance nutrient use efficiency. Slow-release fertilizers (SRFs) offer a promising approach to more sustainable nutrient management by gradually releasing nutrients in alignment with crop growth stages. This strategy minimizes nutrient losses and reduces the frequency of fertilizer applications [34,35].

Slow-release fertilizers (SRFs) can be categorized into three main classes: coated SRFs, matrix-based SRFs, and chemically bound SRFs, based on the mechanisms that regulate nutrient release [36].

Coated SRFs control nutrient release through an impermeable or semi-permeable film, with nutrients gradually diffusing through the coating over time [37,38], which reduces nutrient losses due to volatilization, leaching and runoff [39,40]. They are typically classified into three main categories based on the nature of the coating materials: inorganic mineral-based coatings (sulfur, phosphates), synthetic polymer-based coatings (such as PVDC copolymers, polyolefins, polyurethane, urea-formaldehyde resin, polyethylene) and bio-based or biodegradable coatings (including starch derivatives, cellulose, lignin, chitosan, natural rubber, guar gum and fatty acid salts) [35,41]. Nutrient release rate tends to be relatively constant over time, governed by the coating’s thickness and chemical composition [42]. Despite their benefits, coated SRFs face challenges, including the accumulation of non-biodegradable polymers in soil (up to 10%) [43] and high production costs that limit adoption by small-scale farmers [39,44].

Matrix-based SRFs, in contrast, do not rely on an external barrier but regulate nutrient release through physical entrapment, adsorption or diffusion within a solid matrix [36]. Among matrix-based SRFs, hydrogel-based systems use crosslinked water-absorbing polymer networks (synthetic polyacrylates or bio-polymers like starch, chitosan, alginate) to entrap nutrients within a swollen matrix and gradually release them [45]. For example, the literature has reported that a case of a hydrogel-based SRF composed of hydroxypropyl methyl cellulose, polyvinyl alcohol, glycerol and blended paper achieved 87.0% urea release over 44 days in soil compared with 97% release in only 4 days for the same amount of pure urea [46]. Release from SRF hydrogels typically follows a three-stage process. In the initial lag phase, the hydrogel absorbs water and swells, with minimal nutrient release. This is followed by a constant release phase, through diffusion in the polymer matrix. Finally, in the decay phase, the release rate decreases due to nutrient depletion or environmental changes [47].

Matrix-based SRFs also include much simpler approaches based on natural porous mineral materials, in which nutrients are dispersed within an inorganic matrix [48] (such as zeolites, bentonites, diatomites, pumice or hydroxyapatite clinker), within combined inorganic-polymeric matrices [49], or organic matrices [50], typically through straightforward mixing processes. In inorganic matrix systems, tuning matrix porosity, such as by incorporating clay nanoparticles, can achieve approximately 60–70% nutrient release over a 30-day period by controlling adsorption, ion exchange and physical entrapment within the porous structure [51]. Similarly, an organic matrix SRFs formulated from agricultural residues (cow manure, clay, neem, rice bran, gum arabic) released ammonium slowly over 50 days [50].

A third category of SRFs includes materials in which nutrients are chemically bound, such as struvite, bone meal or blood-derived products, whose release is governed by microbial and chemical degradation rather than simple diffusion or desorption [36]. These systems do not require advanced chemistry or synthetic coating and can be obtained at low cost. Moreover, using materials commonly regarded as waste contributes to resource efficiency, aligning with circular economic principles.

Numerous studies highlight the potential of recovered materials to produce slow-release fertilizers (SRFs), emphasizing the environmental and economic advantages of such approaches. Different studies explored the production of SRFs from byproducts such as leonardite and zeolite-rich scraps and rice straw with promising applicability in agriculture [52,53]. Likewise, from different studies, it emerged that struvite, bone meal and animal blood are rich sources of nitrogen and phosphorus, but their actual availability is constrained by technical and regulatory factors. For example, struvite is a precipitated mineral (MgNH_4_PO_4_·6H_2_O) that can be recovered from municipal wastewater or sewage sludge [54,55]. Unlike conventional mineral phosphates used in agriculture, struvite has low water solubility, allowing for the gradual release of nutrients into the soil. Recovery studies suggest that European sewer systems contain enough phosphorus to potentially replace 40–50% of the mineral phosphorus required for fertilization, highlighting a significant recycling potential [56,57,58], and the literature shows that the environmental risks associated with using precipitated struvite as a renewable phosphate fertilizer are often deemed as insignificant [59]. Although struvite production is not yet sufficient for meeting the total phosphorus demand in agriculture, its production trend is steadily rising, as demonstrated by the increasing number of commercial-scale plants currently in operation or under development [60,61,62].

Blood and bone meal were, among other organic ingredients, utilized as natural SRFs, since they are able to gradually release nutrients during their degradation process [63]. In the EU, the annual production of animal byproducts (ABPs) is nearly 20 million tons [64,65]. Specifically, the EU is estimated to generate over 4 million tons of animal bone biomass each year, which contains approximately 85–88% of the total phosphorus found in vertebrate bodies [66]. While this availability is significant, its use is subject to strict regulations, as category 3 materials (from healthy animals) are permitted for use as fertilizers only after undergoing treatments such as sterilization under heat and pressure (this inevitably led to higher prices for these materials). In practice, a large amount of the animal byproducts that could potentially be used are incinerated, resulting in the loss of valuable nutrients [65,67].

The use of waste materials rich in P, such as struvite [68] or animal bone meal and meat [29,69], also offer a strategy to make up for P-reservoir depletion and geopolitical dependence [70] and close the loop of the P-cycle.

Furthermore, blood typically contains about 13% nitrogen, along with smaller amounts of phosphorus, micronutrients and organic matter [71]. Its nitrogen content mineralizes gradually in the soil, which helps reduce nutrient losses by leaching, while still achieving comparable or even better performances than chemical NPK fertilizers [72,73]. From an environmental perspective, using animal blood as fertilizer offers a way to valorize a byproduct of the meat industry that would otherwise represent a problematic waste stream. Fresh blood accounts for approximately 3–8% of the live weight of slaughtered animals [74]. However, strict sanitary regulations govern the treatment and use of blood products [75]. In summary, while the technical availability of animal blood as a fertilizer is substantial, it remains largely underutilized. From an environmental standpoint, reusing animal-derived materials such as meat and bone meal as fertilizers can be a more sustainable option than disposal. A life cycle assessment (LCA) showed that their agricultural application reduces greenhouse gas emissions and acidification compared with incineration [76].

In our work, building on previous experience in the field [77,78,79,80,81,82], we developed waste-based N-P-K slow-release fertilizers, exploiting byproducts from the animal processing chain (bone meal and bovine blood) as sources of macronutrients. Pumice scraps from the mining industry were also incorporated with the intention of obtaining lightweight granules with water retention capacity. The goal was to obtain a product that is scalable, cost-effective and environmentally sustainable. Additionally, while current research on SRFs primarily focuses on the slow release of individual nutrient elements [41], this study developed a product containing all the essential macronutrients required for plant growth. The granules were obtained through a physical process by mixing the previously ground precursors using a liquid binder (liquid blood or calcium lignosulphonate). The rationale was to use materials in which the nutrients are not readily available, being in a complex organic form or inorganic insoluble form in water, but soluble in acidic and metal complexing solutions, mimicking the behavior of soil’s organic substances [83]. However, to have a small immediate release of nutrients to support initial plant development, a small quantity of KNO_3_ was added as a source of nitrogen (N) and potassium (K). Nutrient release was assessed in the laboratory under different extractive conditions (2% citric acid, acetic acid 0.5 M) up to 90 days. Additionally, the performances of three different granules were tested on specimens of lettuce.

The present study aims to explore the potential of waste-derived materials, specifically animal blood, bone meal and pumice scraps, as a basis for the development of sustainable slow-release NPK fertilizers. Rather than providing an exhaustive evaluation of agronomic performance, the focus is on selecting and characterizing the most promising formulation to be advanced in future studies.

To this end, several granulated formulations were produced and evaluated in terms of physical and chemical characteristics, nutrient content, and release kinetics under acidic conditions that simulate both rhizospheric activity and environmental leaching scenarios. Preliminary greenhouse trials on lettuce were also carried out to rule out phytotoxicity and assess short-term plant response and support the optimization of the formulations.

The underlying hypothesis is that combining organic and mineral waste components in a controlled matrix can lead to fertilizers that not only provide gradual nutrient availability and contribute to soil fertility and structure but also contribute to the circular use of underutilized resources.

This initial phase is intended to lay the groundwork for subsequent investigations focused on long-term agronomic behavior, in-soil nutrient dynamics and the economic viability of scaling up production.

## 2. Materials and Methods

### 2.1. Materials

Waste-based fertilizers (WBFs) were synthesized through the granulation of byproducts and, to a small extent, raw materials. The procedures will be discussed more in depth in Section 2.2.1.

The granules obtained (codified as WBF in Table 1) are composed of three natural materials and one of chemical grade. The first material is characterized as pumice scraps, the second one consists of bone meal (main P-source), and the third is animal blood, either liquid or dry (N-source). The raw animal-derived materials (liquid and dry blood and bone meal) were industrially sterilized using heat treatment to ensure safety from a health and phytosanitary point of view, in accordance with the provisions of the EEC Health Regulation 1069/2009 [75], designed to guarantee the elimination of all pathogenic agents (bacteria, bacilli, various viruses, etc.). We also added a quantity (<10% *w*/*w*) of KNO_3_ (ACS grade) to have a fast-release component. A lignosulfonate binder was used for the granulation process when liquid blood was replaced with dry blood. Lignosulfonate, for its characteristics, is one of the best binders for pelleting feed and other livestock applications [84]. When lignosulfonate salts are added to synthetic inorganic fertilizer solutions, the effect is the hardening of the final product and the reduction of particle caking. Caking problems, namely the formation of lumps or agglomerates during storage, negatively affect the physical properties of fertilizer during storage, potentially affecting the release of nutrients required by the soil [85].

Other materials used for the following tests include citric acid (≥99.5%, ACS grade), acetic acid (100%, Suprapur^®^), nitric acid (65%, Suprapur^®^), sulfuric acid (96%, Ultrapur^®^), sodium hydroxide (≥99%, ACS grade), sodium molybdate dihydrate (≥99.5%), ammonium molybdate (99.98% trace metals basis), quinoline (reagent grade, 98%) and dipotassium phosphate (ACS reagent, ≥98%), which were supplied by Sigma-Aldrich. Kjeldahl Selenium Catalyst Tablets, Technical, were acquired from Fisher Chemical™, Thermo Fisher Scientific, Waltham, MA, USA.

### 2.2. Synthesis and Waste-Based Fertilizers Characterization

#### 2.2.1. Sample Preparation

**Figure 1:**
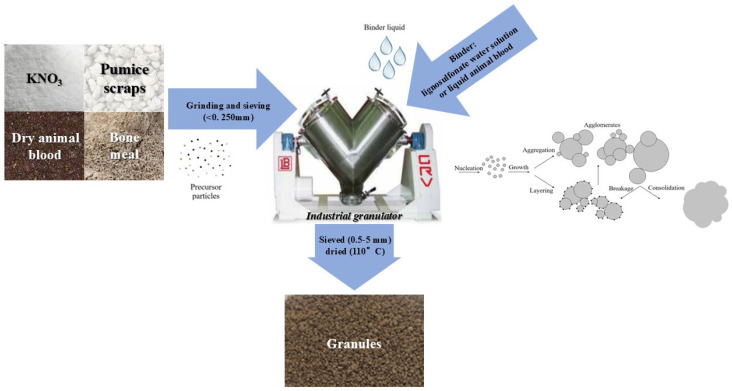
Schematic representation of the industrial wet granulation process. Granule formation occurs through successive mechanisms: nucleation, particle growth, layering and aggregation, followed by consolidation. Occasional breakage of agglomerates can also occur during the process.

The procedure includes characterization and preparation by grounding and sieving (<0.250 mm) of three materials that were later mixed and granulated through an industrial granulator (GRV7 Model, provided by LB Offcine Meccaniche S.p.A., Fiorano Modenese, MO, Italy) in sequential steps. Preliminary wet granulation trials (WBF1–3) identified the <0.250 mm powder fraction as the optimal size, as it promoted efficient nucleation and granule growth, as well as granule cohesion and structural integrity, with reduced binder and water quantity. The process of wet granulation is depicted in Figure 1.

When liquid blood was used as a source of N, no additional binder was needed for the granulation process, as the moisture of the sample was sufficient. On the other hand, when using dried blood, calcium lignosulfonate was necessary. The granules were sieved to a 0.5–5 mm size range and dried at 110 °C (residual humidity < 1.0% *w*/*w*). The material ratios for formulations WBF4 (liquid blood-containing), WBF5 and WBF6 (dry blood-containing), produced using this method, varied from 33 to 38% (*w*/*w*) of pumice scraps, from 54 to 58% (*w*/*w*) of animal blood and bone meal and from 5 to 9% (*w*/*w*) of KNO_3,_ being WBF6 the formulation with the lowest KNO_3_ content. The nominal NPK content is reported in Table 1, where are also showed all the formulations studied.

The WBF4 formulation was optimized to achieve the right moisture content to obtain the granules, following adjustments made in response to issues identified in three preliminary tests (WBF1, WBF2 and WBF3, not detailed here as their solid phase compositions are identical to those of WBF4). Specifically, WBF4 was refined to address odor issues related to liquid blood content, WBF5 was adjusted to reduce phytotoxicity from excessive nitrogen (more details in Section 2.5*)*, and WBF6 represents the optimal outcome, balancing odor management with a more gradual nitrogen release. Therefore, the three formulations WBF4, WBF5 and WBF6 discussed here are the result of a design optimization process targeted at addressing the challenges encountered during the experimental trials.

#### 2.2.2. Raw Materials Characterization

Early formulations were designed to achieve the minimal nutrient content required by European regulation on NPK fertilizers [86], so that the content of each nutrient is at least 5% (*w*/*w*).

Elemental analysis and X-Ray Fluorescence (XRF) were performed on the precursors to evaluate NPK content. N content is expressed as total N % (*w*/*w*), P content is expressed as P_2_O_5_% (*w*/*w*), and K content is expressed as K_2_O % (*w*/*w*), as indicated by regulation. Digestion with concentrated H_2_SO_4_ solution in Kjeldahl digestion unit with selenium catalyst tablets at 400 °C, followed by determination by distillation (Kjeldahl method), was performed to assess the content of ammonium (N-NH_4_^+^) and organic nitrogen.

Loss on ignition (L.O.I.) and ash content determination were performed through combustion at 950 °C for 2 h using an electric muffle furnace. Humidity was assessed by heating at 105 °C for 2 h. Evaluating the precursor’s humidity was specifically crucial to the granulation process. The results are expressed in percentage % (*w*/*w*). Specific surface area (SSA) was calculated using Brunauer–Emmett–Teller (BET) analysis. Characterization of the precursors is reported in Table 2.

#### 2.2.3. WBFs Characterization

**Figure 2:**
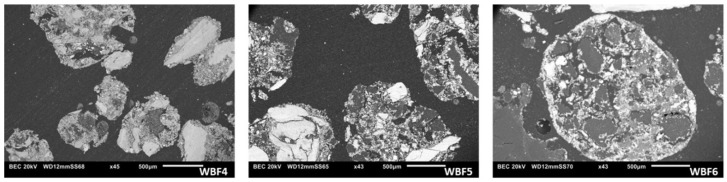
Scanning Electron Microscope (SEM) images of WBF4, WBF5 and WBF6 granules.

WBFs synthesized were fully characterized, and the physical parameters of the granules were examined. Humidity and L.O.I. were measured as defined above. Specific surface areas were determined by BET analysis, while the granule morphology was qualitatively assessed using Scanning Electron Microscopy (SEM) images. More in detail, the homogeneous distribution of various components within the WBFs granules, which have a rounded shape, is shown in Figure 2. The darker particles are primarily composed of carbon and originate from the blood component. These dark particles are not clearly visible in WBF4, as it contains liquid blood that is homogeneously distributed throughout the granule.

Nitrogen as ammonium (N-NH_4_^+^) and organic nitrogen contents in fertilizers were determined using the Kjeldahl method after digesting the samples with concentrated sulfuric acid with selenium tablets; thus, the acid-digested samples were distilled to produce ammonium. Total nitrogen was obtained through elemental analysis. Finally, nitrogen as nitrate (N-NO_3_^−^) was obtained by subtracting the contents of N determined by the Kjeldahl method from the total nitrogen content. The total content of P and K was determined by XRF analysis on the granules. The fraction of P soluble in mineral acids (P_2_O_5_^MA^) was determined by digestion in nitric–sulphuric acid mix, followed by the gravimetric method with quinoline phosphomolybdate. Table 3 lists the primary characteristics, concentrations, and pools of NPK of WBFs samples.

The SSA in formulation WBF4 is significantly lower than in WBF5 and WBF6, probably due to the liquid blood content, which, when dried, can cover the particles and reduce porosity. The SSA values for WBF5 and WBF6 are also much lower when compared to pure pumice (Table 2); this behavior can be attributed to the wet granulation processes, whereby fine particles of other materials can partially close the porosity of the material.

The tests regarding the performance of the WBFs (kinetics of nutrient release, agronomic performances and antimicrobial properties) were carried out only on formulations WBF5 and WBF6, as WBF4 was deemed unsuitable for large-scale production due to the challenges in managing its odor during the granulation process.

### 2.3. Weight Loss in Water

To preliminarily assess the performance of the formulations as slow-release fertilizers, water solubility tests were conducted. Exactly 1 g of each WBF was weighed using a four-decimal precision laboratory balance and placed into a 50 mL screw-capped polypropylene bottle. Millipore water was then added to a final volume of 10 mL.

The samples were maintained under static conditions at room temperature for predetermined interaction times: 0.5 h (30 min), 2 h, 4 h, 168 h (7 days), 336 h (14 days), 504 h (42 days), 672 h (90 days). A separate sample was prepared for each time point. After the designated contact period, the samples were quantitatively filtered using paper filters. The recovered solid residues were dried at 105 °C for 24 h and subsequently weighed. The mass loss, expressed as a percentage of the initial sample weight, was used to assess the solubility of the formulation over time.

### 2.4. Kinetics of Nutrient Release

The release kinetics of N-P-K nutrients from the two WBFs were assessed using 2% citric acid (CA) and 0.5 M acetic acid (AA) solutions as extractants, following the specifications outlined in Italian legislations and standards [87,88]. The two extractants were chosen to assess nutrient release for two distinct purposes: CA solubility indicates the fraction of nutrients potentially available to plants, as the chelating properties of citric acid effectively mimic the activity of soil humic substances [89]. Conversely, solubility in AA, a non-chelating agent, provides an indication of the nutrients prone to leaching under acidic conditions, such as those caused by acid rain, due to its low pH. In this context, a slower and more gradual release of nitrogen and phosphorus is desirable for minimizing the potential environmental impact associated with excessive nutrient leaching.

The method to assess the kinetics of release included the following steps: for each extractant and formulation tested, 1 g of fertilizer was deposited in a 100 mL screw-capped polypropylene laboratory bottle. The bottle was then filled with the chosen solution up to 100 mL. An independent sample was prepared for each of the selected contact times (30 min; 168, 336, 504, 672, 1008 and 2160 h). Contact times were chosen to evaluate the WBFs’ slow-release behavior. More specifically, European standards [90] require that the total NPK release should not exceed 15% before 24 h and 75% before 672 h (28 days). The sealed bottles were shaken at room temperature (20 °C) at an agitation of 40 rpm for the specified contact time. The sample was then quantitatively filtered through paper filters.

The pH and electrical conductivity of the filtrate were immediately measured and held at 10 °C until ICP-MS (triple quadrupole detector) analysis to determine the released P_2_O_5_ and K_2_O. ICP-MS tests were conducted within three days of filtration. Filters containing the solid residue were dried at 105 °C for 24 h. The dried residue was weighed with an analytical balance to evaluate mass loss over time, then stored at room temperature for elemental analysis to estimate residual nitrogen content. The same approach was used on blank samples of 100 mL of 2% citric acid or 0.5 M acetic acid solution, which were handled in identical containers and under identical conditions for each contact time.

### 2.5. Agronomic Performances

The agronomic efficiency of WBFs was evaluated through the cultivation of lettuce specimens (*Lactuca sativa* L., cultivar ‘Canasta’) in greenhouse conditions. The test was conducted following the method used to evaluate the depressing or biostimulant effects of composted matrices [91].

The test involved the following experimental treatments: two controls—soil (substrate) unfertilized and a reference system, soil with a commercial NPK fertilizer with a concentration corresponding to 8% (*w*/*w*) N—17% (*w*/*w*) P_2_O_5_—8% (*w*/*w*) K_2_O and applied at 3 g/kg of substrate, and two products (WBF5 and WBF6) applied to the soil, each tested at three different doses: 1.25, 2.5, 5.0 and 10 g/kg of substrate.

The substrate used for the greenhouse test consisted of a nutrient-poor sandy soil, selected to minimize background nutrient contributions and emphasize the performance of the tested fertilizers. No hydroponic or synthetic model soil was used.

Each treatment included four independent replicates, represented by four pots with a diameter of 8 cm. Three lettuce seedlings at the first true leaf stage were transplanted in each pot. The pots were maintained in a controlled environment with a regulated water supply, with a light cycle of 16 h day and 8 h night. The substrate used was predominantly sandy.

After 21 days, the cultivation was stopped, and the fresh and dry weight (at 75 °C) of the aerial biomass produced by each replicate was measured. Fresh and dry indexes (FI and DI) were calculated, normalizing the mean values of fresh weight and dry weight for each thesis with respect to the value of the control.

To assess potential contamination risks, heavy metal content was also monitored in soil fertilized with WBF6 using ICP-MS (Figure S1).

This test was conceived as a preliminary screening to support the final selection of the most suitable formulation during the development phase. Its aim was not to provide an exhaustive agronomic evaluation but to guide formulation optimization based on early plant response.

### 2.6. Antimicrobial Properties

The antimicrobial properties were evaluated using the Kirby–Bauer method [92,93]. The bacteria used for this test were both Gram-negative (*Escherichia coli*, *Pseudomonas aeruginosa*) and Gram-positive (*Enterococcus faecalis*, *Clostridium perfringens*), incubated in the presence and absence of the samples WBF5 and WBF6. Bacterial pellets were dissolved in distilled saline water (0.9% NaCl) and diluted to obtain bacterial suspensions of 105 CFU/mL, which were then plated on appropriate solid agar media. Specifically, *E. coli* (ATCC 25922) was cultured on Tryptone Bile X-Gluc (TBX) Medium, *C. perfringens* (ATCC 13124) on Tryptose Sulfite Cycloserine (TSC) Agar with D-cycloserine, *P. aeruginosa* (ATCC 27853) on *Pseudomonas* CN Agar, and *E. faecalis* (ATCC 29212) on Slanetz–Bartley Agar Base.

Before incubation, 200 and 300 mg of sample powder were sterilized under UV light for 1 h and placed at the center of Petri dishes (PPDs). The incubation conditions were as follows: *E. coli* at 44 °C for 24 h, *C. perfringens* at 44 °C for 24 h under anaerobic conditions, and *E. faecalis* and *P. aeruginosa* at 36 °C for 48 h. After incubation, the diameters of the inhibition zones were measured and recorded in relation to the 6 cm Petri dish diameter. Four diameter measurements were taken for each sample to calculate the mean and standard deviation.

## 3. Results

### 3.1. Weight Loss in Water

**Figure 3:**
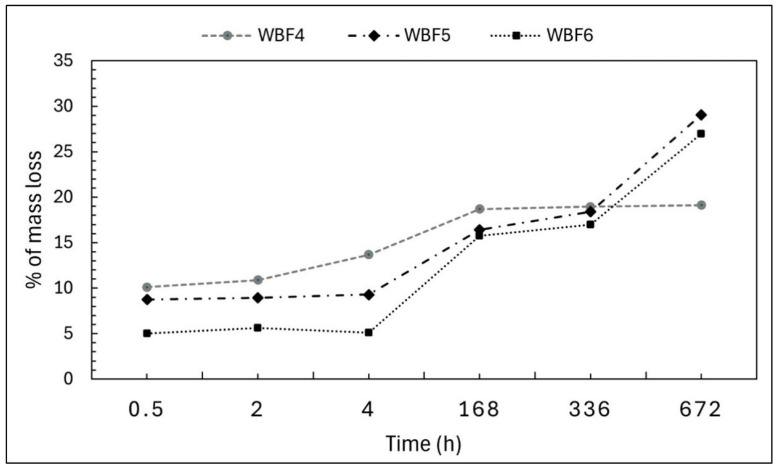
Mass loss in bidistilled water (% *w*/*w*) measured for WBF4, WBF5 and WBF6 over a period of 28 days.

The water solubility of the three formulations under investigation was compared, with slower solubility over time being desirable as an indicator of potential performance as a slow-release fertilizer (Figure 3).

WBF4 exhibited the highest initial mass loss, likely due to its higher liquid (liquid blood) content, being 10% (*w*/*w*) at 30 min, 14% (*w*/*w*) at 4 h and 19% (*w*/*w*) at 168 h. This solubility then stabilized through the 28-day period.

The mass loss profiles of WBF5 and WBF6 were similar. From 30 min to 4 h, the mass loss remained stable at 9 and 5% (*w*/*w*), respectively, with WBF5 showing almost double the loss of WBF6, as expected given its higher KNO_3_ content. Beyond this period, the release rates increased, and the percentage mass loss for WBF6 gradually approached that of WBF5, with both reaching similar values at 168 h—16% (*w*/*w*), 336 h—18% (*w*/*w*) for WBF5 and 17% (*w*/*w*) for WBF6, and 672 h—29% (*w*/*w*) for BF5 and 27% (*w*/*w*) for WBF6.

### 3.2. Kinetics of Nutrient Release

**Figure 4:**
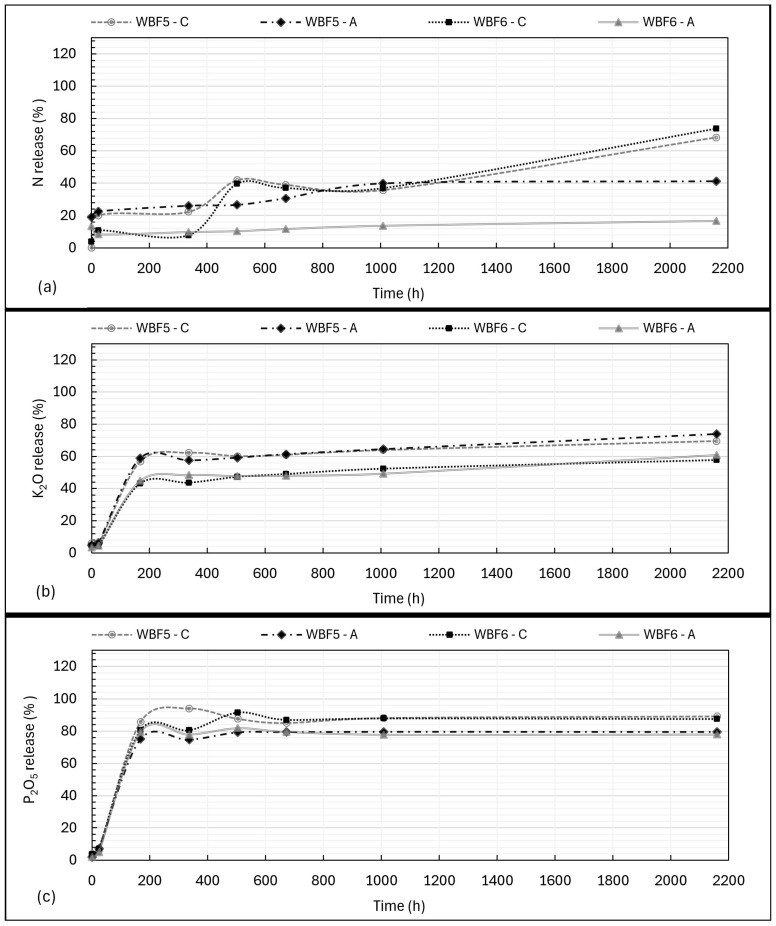
The kinetics of N (**a**), K_2_O (**b**) and P_2_O_5_ (**c**) release in citric acid (C) solution (2%) and acetic acid (A) solution (0.5 M) according to the TNC of NPK in the WBFs.

In this study, the kinetics of release of N-P-K were evaluated at defined intervals, both at short times (0.5 h and 24 h) and longer times until 90 days (specifically 168, 336, 504, 672, 1008 and 2160 h). Results were expressed in % of nutrients (%P_2_O_5_, %K_2_O and %N compared with the total % (*w*/*w*) of each single nutrient in the formulation) released over time. Results are shown in Figure 4.

These percentage were also used to calculate the Total Nutrient Release (TNR_(t)_) at 24, 672 and 2160 h (the latter, the longest contact time, 90 days). Only the release in CA was considered for this calculation, as the chosen solution mimics soil extraction conditions (see Section 2.4). At 24 h, TNR_(t)_ was, respectively, 10% for WBF5 and 8% for WBF6. At 28 days, TNR_(t)_ reached 62% for WBF5 and 57% for WBF6. At 90 days, TNR_(t)_ was, respectively, 75% for WBF5 and 73% for WBF6. TNR_(t)_ was calculated as indicated in Equation (1), while Total Nutrient Content (TNC, % (*w*/*w*)) was calculated as per Equation (2).

Equation (1). Calculation of TNR_(t)_ (%) is performed at a definite time (t).

(1) $${TNR}_{\left(t\right)}=\left\{\left[rN_{\left(t\right)}\cdot \frac{\%(N)}{TNC}\right]+\left[{rK}_{2}O_{\left(t\right)}\cdot \frac{{\%(K}_{2}O)}{TNC}\right]+\left[rP_{2}{O_{5}}_{\left(t\right)}\cdot \frac{\%{(P}_{2}O_{5})}{TNC}\right]\right\}\cdot 100$$

Equation (2). Total Nutrient Content (TNC) at the initial time in the formulations is calculated from the experimental nutrient % (*w*/*w*), as expressed in Table 3.

(2) $$TNC=[\%(N)+{\%(K}_{2}O)+\%{(P}_{2}O_{5})]$$

%(N), % (P_2_O_5_), % (K_2_O) = initial % (*w*/*w*) of N, P_2_O_5_, and K_2_O in the fertilizer.
rN_(t)_, rP_2_O_5(t)_, K_2_O_(t)_ = fractions (%) of N, P_2_O_5_, and K_2_O that have been released at time t, with respect to the initial content (% (*w*/*w*)) of each single nutrient.
TNC = Total Nutrient Content

The release of phosphorus (P_2_O_5_) and, more evidently, nitrogen (N) was generally higher in citric acid compared with acetic acid, particularly over extended periods. Potassium (K_2_O) release, however, appeared to be independent of the extractant solution.

The release of nitrogen (N) in citric acid and acetic acid differs not only in the total amount released but also in the release dynamics. In AA, most of the nitrogen from KNO_3_ is extracted within the first 0.5–24 h, with 22% of the total N released from WBF5 and 8% from WBF6. By the end of the experiment, 41% of N had been released from WBF5, and only 16% from WBF6.

In contrast, the release of nitrogen for both formulations in CA reaches similar values to those of WBF5 in AA in the initial phase (up to 1008 h, 42 days), albeit with some fluctuations likely due to dissolution and precipitation phenomena. However, it exhibits a more consistent release over extended periods. By the conclusion of the 90-day study, total nitrogen release reached 68% for WBF5 and 74% for WBF6, maintaining a steady release rate from 42 to 90 days.

**Figure 5:**
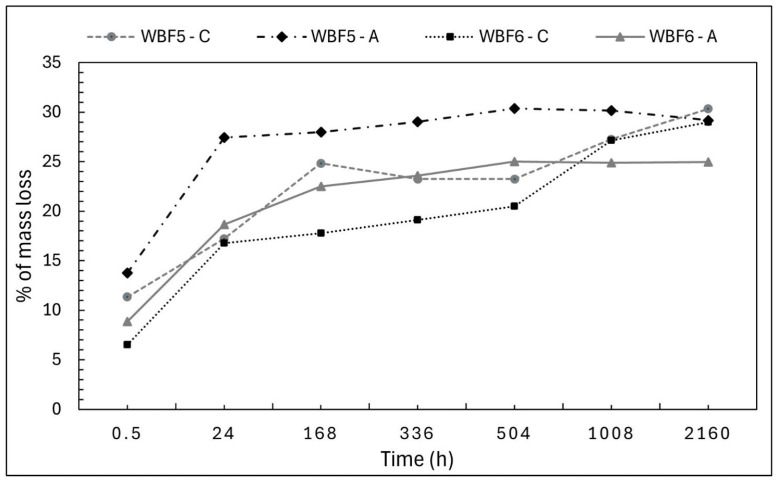
Monitoring of the % (*w*/*w*) mass loss in citric acid (C) and acetic acid (A) of WBF5 and WBF6.

Mass loss and pH were monitored over time for both extractants, revealing distinct trends (Figure 5). In solution AA, the mass loss profiles of WBF5 and WBF6 are similar, with WBF5 exhibiting higher values, which was expected due to the higher content of KNO_3_. The most significant loss occurs within the first 24 h, reaching 27% (*w*/*w*) of the total weight for WBF5 and 25% (*w*/*w*) for WBF6. After this initial phase, the release rate stabilizes, remaining almost constant from 1 to 90 days.

In CA, mass loss profiles are more dynamic. For WBF5, a rapid increase occurs between 0.5 and 168 h, reaching 24% (*w*/*w*), followed by a slower but steady increase to 30% (*w*/*w*) at 2160 h. WBF6 shows a slower initial increase, reaching 19% (*w*/*w*) in 24 h, 20% (*w*/*w*) at 504 h, and 29% (*w*/*w*) by the end of the 90-day period. Unlike the AA extraction, the mass loss values for WBF5 and WBF6 demonstrated an approximate convergence over a longer period; this behavior provides clear evidence that chelation mechanisms are necessary for solubilizing a portion of the granules in WBF6.

**Figure 6:**
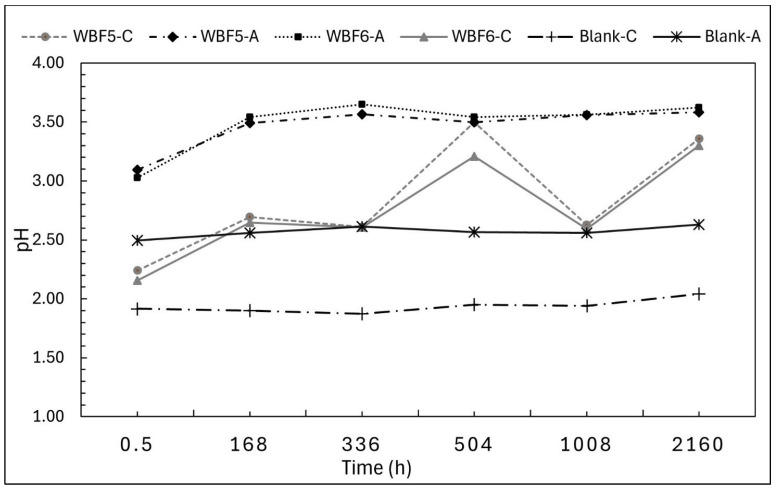
pH of the filtrated solutions of WBF5 and WBF6 in extractant citric acid 2% (C) and acetic acid 0.5 M (A). The pH of blank solutions was also monitored.

The monitoring of eluate pH over time (Figure 6) revealed an increase compared with the reference (blank solutions).

Like the mass loss trends, the pH in acetic acid (AA) rises rapidly during the first 168 h and then stabilizes at 3.6, maintaining this value until the end of the 90-day period. In citric acid (CA), the pH increase is more gradual within the first 7 days. Subsequently, the pH profiles of WBF5 and WBF6 both fluctuate between 2.7 and 3.5 over the remaining period.

**Figure 7:**
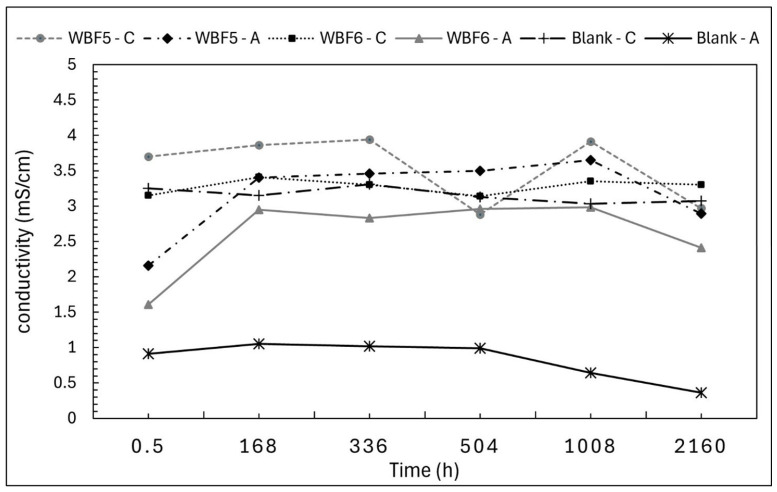
Conductivity of the filtrated solutions of WBF5 and WBF6 in extractant citric acid 2% (C) and acetic acid 0.5 M (A). Conductivity of blank solutions was also monitored.

Conductivity data show coherent behavior with pH measurements (Figure 7).

### 3.3. Agronomic Performances

Preliminary tests for assessing the agronomic performances of WBFs were carried out on WBF5 and WBF6 in two separate periods of time. The primary objective was to assess whether the tested formulation could enhance biomass production compared with a commercial fertilizer and to evaluate any potential phytotoxic effects in relation to unfertilized control. In this context, the “substrate” refers to the soil used in all treatments, which also serves as the unfertilized control (without any added fertilizer). The normalized fresh and dry weight indices provide a straightforward means of highlighting both the agronomic performance and the absence or presence of phytotoxicity.

**Figure 8:**
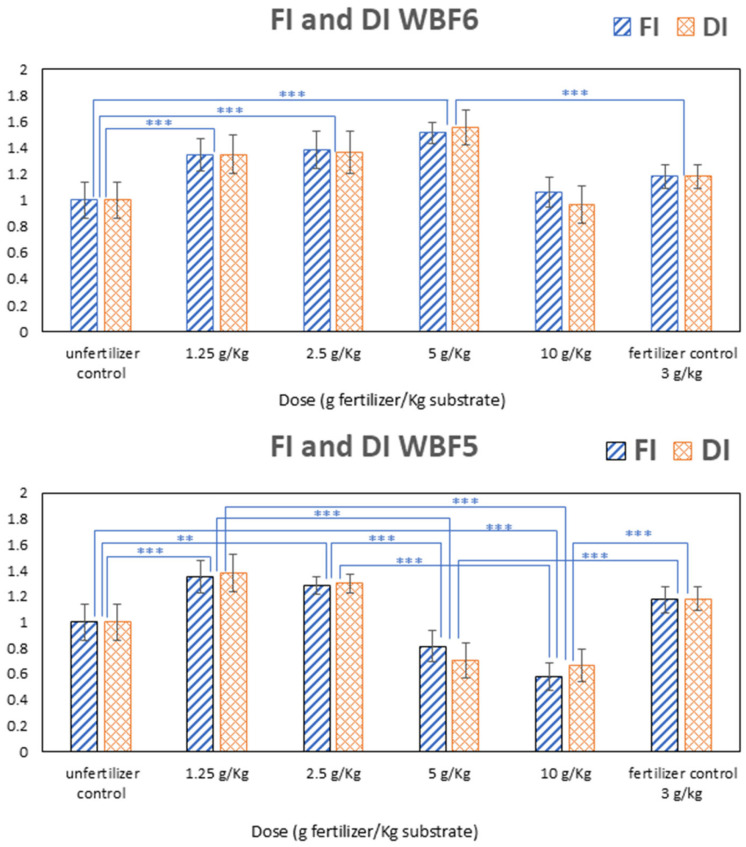
Biomass production: fresh index (FI) and dry index (DI) of WBF6 and WBF5. Data are means of five replicates, and the error bars represent the standard deviation. Multiple comparisons were performed with a Tukey–Kramer test. Symbols ** and *** indicate significance at *p* < 0.01 and *p* < 0.001, respectively.

Results are presented in Figure 8, where values have been normalized to the unfertilized control (soil alone) and compared to those obtained using a commercial fertilizer (fertilized control) applied at a standard rate (3 g/kg of substrate).

At its minimal application rate (1.25 g/kg of substrate), WBF5 significantly enhanced biomass production, leading to an increase of +36% and +15% compared with the unfertilized control and fertilized reference, respectively. However, increasing the dose of application resulted in a decreased biomass production both with respect to the control and the reference: dose 5 g/kg of substrate yielded (−19%) compared with the sole soil and (−31%) in comparison to the reference. The higher dose (10 g/kg of substrate) showed a halving of biomass production (−50%) compared with the commercial fertilizers. One hypothesis posited that the high content of the immediately soluble component (KNO_3_) in the formulation led to an overload of nitrogen during the development of the specimens, consequently diminishing biomass production. It is well-known that nitrogen is crucial for plant growth. Nitrogen significantly affects crop yield and quality, supports vegetative growth and is essential for the development of reproductive organs. In contrast, nitrogen deficiency can lead to reduced photosynthesis and stunted growth, impacting yield and quality. However, even excessive nitrogen application can have negative consequences on plant health, inhibiting root development and decreasing plant biomass [94] and leading to decreased plant vigor and productivity.

Consequently, WBF6 was formulated with a 50% reduction in KNO_3_ content to ensure a more measured supply of bioavailable nitrogen early in the plant life cycle. When repeating the same agronomic performance tests on this formulation, we found that at the minimal application dose (1.25 g/kg of substrate), the increase in biomass production was comparable to that of WBF5 at the same dose. Both formulations showed a similar increase relative to the control (+34%) and the reference (+14%). At doses of 2.5 and 5 g/kg of substrate—both lower and higher, respectively, compared with the control dose of 3 g/kg—agronomic performance continued to improve. Specifically, dose 2.5 g/kg yielded (+38%) in comparison to the control and (+17%) in comparison to the fertilized reference and (+50%) and (+28%) at dose 5 g/kg. Incrementing further the dose to 10 g/kg led to a decrease in the agronomic performances compared with the fertilized reference (−10%), but the formulation still yielded better results in comparison to the unfertilized control (+6%).

Growth indices (GI) are calculated as follows in Equation (3).

Equation (3) Calculation of the growth indices (fresh and dry) for a given dose of fertilizer after 21 days. Growth index quantifies the increase in foliar biomass due to fertilizer application compared to an unfertilized reference.

(3) $$GI=\frac{m_{d}}{m_{r}}\cdot 100$$

GI = growth index (%).
m_d_ = average measured weight of foliar biomass after 21 days for plants given a specific dose of fertilizer
m_r_ = average measured weight of foliar biomass after 21 days for the unfertilized reference.

### 3.4. Antimicrobial Properties

**Figure 9:**
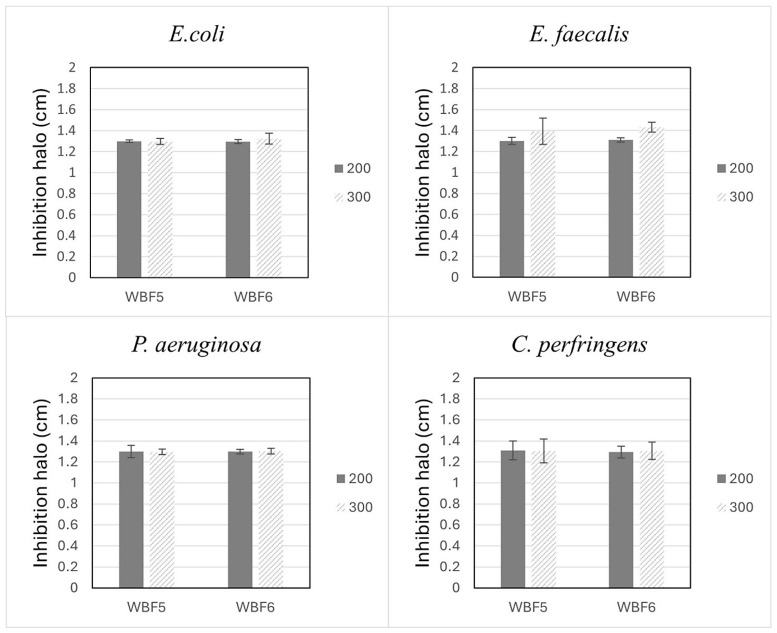
Antimicrobial activity of WBF5 and WBF6 against *E. coli*, *E. faecalis*, *C. perfringens*, and *P. aeruginosa*.

This study assessed the antimicrobial properties of WBF5 and WBF6. Both samples exhibited antibacterial activity against microbes such as *E. coli*, *E. faecalis*, *C. perfringens* and *P. aeruginosa*. As indicated in Figure 9, all the samples showed an inhibition halo of 1.3 ± 0.2 cm for all the tested bacteria. Although the samples were tested at two different quantities, 200 and 300 mg, the data did not indicate a dose-dependent response.

The antibacterial efficacy of WBF5 and WBF6 can be attributed to a combination of physical and chemical mechanisms. These samples contain pumice, known for its high porosity and medium to high content of Al_2_O_3_ and Fe_2_O_3_. Such elemental composition and structural properties can perturb ionic balances, interfere with bacterial osmoregulation and lead to the production of reactive oxygen species (ROS). Additionally, the presence of animal bone meal releases calcium and phosphate, impacting bacterial cell osmotic balance. Potassium nitrate, another component, has strong antimicrobial activity by generating reactive nitrogen species that are toxic to bacteria [95].

Furthermore, the lignosulphonate binder in these samples may introduce phenolic compounds, noted for their antibacterial properties [96]. These diverse mechanisms likely work synergistically, enhancing the overall antimicrobial action observed.

## 4. Discussion

Based on both nutrient release study and agronomic performances, WBF6 was identified as the best-performing system, featuring an organo-mineral waste-derived matrix that plays a central role in regulating nutrient release. This formulation demonstrated a cumulative release ranging from approximately 8% after one day to 73% after 90 days.

This extended nutrient release suggests the ability of the formulation to support plant growth for at least three months with a single application. A comparison with a recently studied multi-nutrient SRF based on animal-origin waste (mussel shell powder loaded with cow urine, leading to struvite formation) showed that the cumulative release of macro- and micronutrients was limited to 50% over one month; however, the release was not investigated beyond this time frame [97].

Despite the variations in release dynamics, the Total Nutrient Release (TNR_(t)_) calculations confirmed that both WBF5 and WBF6 meet the criteria for slow-release fertilizers under European regulations and align with values reported for other slow-release formulations in the literature [51,90]. Moreover, nutrient release in acetic acid was overall lower than in citric acid, highlighting that the structure of the formulations is less susceptible to acidification in the absence of chelating capacity. This suggests a limited risk of undesired leaching under environmental changes such as acid rain events.

For individual nutrients, the expected differentiated release profiles, considering the extraction media, were confirmed. Nitrogen exhibited the overall more gradual release profile. As anticipated, an initial phase of rapid release corresponds to the solubilization of KNO_3_ within the first few hours, followed by a gradual increase over time, where the blood-derived fraction, composed primarily of proteins and amino acids, releases nitrogen much more gradually up to 90 days, with the trend still rising at the end of the test period. A recent study developed an N–P–K biochar-based SRF reinforced with hydrotalcite and starch and characterized nutrient release in soil over 30 days. The results showed differentiated release dynamics as well: nitrogen peaked at 45%, with all nutrients displaying plateau behavior; phosphorus exhibited a slow release and reached a maximum of only 13%, while potassium followed a more consistent gradual release, reaching between 45% and 80% depending on the formulation, with an upward trend still observed at 30 days [98].

Agronomic literature indicates [99] that protein nitrogen requires microbial transformation before becoming available to plants: bacteria and fungi must first convert these nutrients into inorganic forms, leading to a slower release. Studies have shown that about 66% of the total nitrogen in protein-rich sources such as blood is mineralized in soil over eight weeks [100], faster than the release dynamics observed in our study in citric acid solution.

A comparison with the literature reveals that performances from different SRF systems can match those of our formulations in terms of nitrogen release profiles and rates. For example, a study on polymer-coated urea (PCU) fertilizers reported a release curve with an initial slow-release phase during the first month, followed by a more accelerated release between 40 and 90 days, ultimately reaching similar total nitrogen release values (about 70–80%) at 90 days [101]. This study was performed under laboratory conditions (in water at 25 °C). It is also known that blood meal (dry blood) is largely insoluble in water, with nitrogen bound in protein structures, but its nitrogen fraction can be solubilized better under acidic conditions [102,103]. In greenhouse tomato trials using blood meal and silage as fertilizers, the application of citric acid via irrigation increased nitrogen (and phosphorus) uptake by plants [104]. Moreover, the same study reported that nitrogen release in soil was further slowed down compared with aqueous media (field vs. laboratory conditions), a trend likely applicable to our SRFs as well.

This comparison suggests that the prolonged nitrogen release observed in our systems is attributable not only to the intrinsic properties of the nitrogen-rich materials (such as blood and bone meal) but also to the porous and “entrapping” structure of the granules. In fact, the granular structure likely slows down hydration, degradation and nutrient migration.

Overall, many recent studies characterize slow-release behavior for only up to 30 days. While this duration is sufficient for demonstrating a delayed nutrient release, many crop species require nutrient support over a longer period. For example, a recent study developed polymer-coated urea formulations based on waste sulfur and myrcene; the best-performing formulation released over 80% of nitrogen within one month in distilled water. In contrast, our WBF6 system reached a comparable nitrogen release level more gradually, over a three-month period [105]. Another study on coated urea granules (using bentonite and carnauba wax) characterized urea release in soil over 60 days. However, most formulations either reached 100% release before the 30-day mark or exhibited an initially slow release followed by a sharp increase, eventually reaching 100% within a few days, which could be compatible with coating failure or granule rupture. Only one formulation exhibited a gradual nutrient release, reaching 100% over approximately 45 days [106]. Recent studies on nutrient-loaded hydrogels have, in some cases, reported less favorable nutrient release performance than the formulations developed in this work. For example, a polysaccharide-based composite gel released 80–95% of nitrogen within just 30 days [107]. Furthermore, various biochar-based slow-release fertilizers (SRFs) demonstrated comparatively inferior performances relative to WBF6. These include a fertilizer consisting of ammonium sulfate adsorbed onto biochar, a slow-release formulation produced by mixing dry urea with activated biochar, and a biochar-based SRF incorporating KH_2_PO_4_ and KNO_3_ [108]. From a technological standpoint, our system is considerably simpler than hydrogel or coating-based SRFs, while still offering comparable or even superior performance in terms of N-release in some cases.

Regarding the release behavior of potassium and phosphorus in citric acid, our formulations demonstrated a less gradual release profile. Potassium continued to be released over the 90-day period, although its release rate declined sharply after 7 days in both citric and acetic acid media. However, normally, an even faster release would be expected, as potassium nitrate, the primary soluble potassium source in our formulations, typically dissolves in a few hours in water, suggesting that the matrix structure contributes to a slower release. In fact, while nitrogen from KNO_3_ was rapidly released within the first few hours, potassium from the same salt exhibited a noticeable delay, suggesting a specific retention mechanism for K^+^ ions, likely due to the pumice porous structure [109].

A portion of K_2_O solubilized from our formulations (about 10% in both formulations) originates from a less soluble source, pumice, where potassium is structurally incorporated within the aluminosilicate matrix. Literature indicates that potassium can be released, albeit slowly, from such insoluble sources. For example, the exposure of K-bearing silicate rocks to 2% citric acid resulted in the release of 2.3% of total potassium after 72 h [110]. Additionally, a previous study on sustainable SRFs used 2% citric acid to evaluate the release behavior of sparingly soluble potassium, reporting 28-day leaching rates of 47.07% in water compared with 85.86% citric acid, highlighting the acid’s ability to enhance potassium mobilization [111].

Phosphorus, on the other hand, reached a near-plateau within 7 days of contact. The phosphorus in our formulation originates primarily from hydroxyapatite (HA) within bone meal. HA is generally considered a slow-release source of P because, like immobilized phosphorus in soils, its solubility is low at neutral-alkaline pH and increases progressively under acidic conditions. The comparatively rapid release documented in our tests can be attributed to the low pH of the extracting solution. Although this facilitated dissolution, it does not directly translate to real soil conditions. In a natural soil environment, pH rarely drops as quickly, and the physical properties of soils tend to slow nutrient dissolution [101].

Literature reports confirm that citric acid (CA) is much more effective than neutral extractants in mobilizing immobile phosphorus sources such as hydroxyapatite [112,113,114]. This is due not only to the acidifying effect of CA, but also to its strong chelating action on Ca^2^^+^ (and other cations), which destabilizes the apatite structure and facilitates phosphorus release [113]. These studies indicated that 77 to 82% of total phosphorus mobilization from hydroxyapatite occurs within the first 24 h when citric acid is used as an extractant. In contrast, our fertilizers achieved similar levels of phosphorus release only after 7 days, indicating a delayed release compared to expectations given these aggressive leaching conditions. Moreover, pH reduction in soils is typically a gradual process, often mediated by phosphate-solubilizing bacteria (PSB), which secrete organic acids capable of mobilizing soil-bound phosphorus over time [115,116]; therefore, it is reasonable to assume that under real conditions (in soil), the P-release from our systems would likely be more gradual.

Even though the release profiles of phosphorus and potassium in our study were less gradual than desired, comparison with literature suggests that, under real-use conditions (in soil), nutrient release would likely be more prolonged. Moreover, agronomic trials conducted under real soil conditions over 21 days already demonstrated that WBF6 sustains nutrient supply effectively, outperforming the unfertilized control. Ongoing long-term trials are currently evaluating WBF6 agronomic performance over a six-month period to better assess nutrient availability across extended timelines. Future work will focus on studying in-soil kinetics and speciation of nutrients, accounting for key soil-related factors such as microbial activity, gradual pH shifts, and physical constraints to diffusion. These insights will be crucial for optimizing formulation design and further characterizing the slow-release behavior.

## 5. Conclusions

Beyond technical aspects, considerations regarding economic feasibility and resource circularity are also essential for future implementation. WBF6 is based on low-cost, recovered materials and avoids the use of expensive and technically complex production methods. Some of these materials (animal blood and bone meal), despite being widely available, remain largely underutilized. Some EU-funded initiatives are already acknowledging the urgent need to transition toward more sustainable economic models. In this context, animal-derived resources such as blood and bone meal hold significant potential as key materials for circular fertilizers [117]. Their utilization aligns with EU directives that recognize the role of animal byproducts in sustainable nutrient management [86]. A sustainable and feasible model could involve the integration of local supply chains to enable nutrient recovery through industrial symbiosis. Looking ahead, dried blood and bone meal could help not only address local phosphorus and nitrogen deficiencies but also reduce reliance on synthetic inputs, contributing to greater strategic autonomy and buffering fluctuations in availability and price linked to global supply chains.

## Figures and Tables

**Table 1:** Theoretical NPK composition of waste-based fertilizers.

Fertilizer	N [% *w*/*w*]	P—P_2_O_5_ [% *w*/*w*]	K—K_2_O [% *w*/*w*]
WBF4	5.2	8.8	8.1
WBF5	6.2	6.1	7.3
WBF6	5.9	6.4	5.4

**Table 2:** Raw material physical (**a**) and chemical (**b**) analyses of precursors.

(**a**)										
PUMICE SCRAPS	SSA [m^2^/g]		Humidity [% (*w*/*w*)]				L.O.I. [% (*w*/*w*)]			
	20.1		3.2				4.3			
BONE MEAL	SSA [m^2^/g]		Humidity [% (*w*/*w*)]				L.O.I. [% (*w*/*w*)]			
	0.71		8.1				57.3			
LIQUID BLOOD	Ash conten [% (*w*/*w*)]		Humidity [% (*w*/*w*)]				L.O.I. [% (*w*/*w*)]			
	>0.1		73.3				<99.9			
DRY BLOOD	Ash content [% (*w*/*w*)]		Humidity [% (*w*/*w*)]				L.O.I. [% (*w*/*w*)]			
	>0.1		13.7				<99.9			
(**b**)										
PUMICE SCRAPS										
^b^ SiO_2_	^b^ Al_2_O_3_	^b^ P_2_O_5_	^b^ TiO_2_	^b^ Fe_2_O_3_	^b^ Na_2_O	^b^ K_2_O	^b^ CaO	^b^ MgO	^b^ SO_3_	^b^ F
60.5	17.2	0.1	0.4	3.2	2.7	7.5	2.4	0.9	-	-
BONE MEAL										
^b^ SiO_2_	^b^ Al_2_O_3_	^b^ P_2_O_5_	^b^ TiO_2_	^b^ Fe_2_O_3_	^b^ Na_2_O	^b^ K_2_O	^b^ CaO	^b^ MgO	^b^ SO_3_	^b^ F
-	-	17.1	-	-	0.7	0.1	23.7	0.6	0.3	-
^a^ N			^a^ C			^a^ H		^a^ S		
6.76			27.59			4.4		-		
^c^ N (Organic + ammonia)										
6.8										
LIQUID BLOOD										
^a^ N			^a^ C			^a^ H		^a^ S		
12.25			46.17			6.39		0.77		
^c^ N (Organic + ammonia)										
13.9										
DRY BLOOD										
^a^ N			^a^ C			^a^ H		^a^ S		
14.99			53.01			7.09		0.26		
^c^ N (Organic + ammonia)										
14.7										

^a,b,c^ data are, respectively, obtained through elemental analysis, X-Ray Fluorescence and Kjeldahl method. Results are expressed in % (*w*/*w*).

**Table 3:** Composition and physicochemical properties of WBFs.

Fertilizer	N^Total^ [% (*w*/*w*)]	N-NH_4_^+^ [% (*w*/*w*)]	N-NO_3_^−^ [% (*w*/*w*)]	N^organic^ [% (*w*/*w*)]
WBF4	4.8	-	0.4	4.4
WBF5	5.8	-	1.2	5
WBF6	6.1	-	0.6	5.4
Fertilizer	P_2_O_5_^Total^ [% (*w*/*w*)]		P_2_O_5_^MA^ [% (*w*/*w*)]	
WBF4	8.2		8.8	
WBF5	5.9		6.3	
WBF6	5.7		6.1	
Fertilizer	K_2_O^Total^ [% (*w*/*w*)]			
WBF4	8.5			
WBF5	7.3			
WBF6	5.4			
Fertilizer	Humidity [% (*w*/*w*)]	L.O.I. [% (*w*/*w*)]	SSA [m^2^/g]	
WBF4	4.9	37.2	0.9	
WBF5	13.2	47.2	3.5	
WBF6	11.9	45.1	4.1	

## Data Availability

The original contributions presented in this study are included in the article. Further inquiries can be directed to the corresponding authors.

## Supplementary Materials

The following supporting information can be downloaded at: https://www.mdpi.com/article/10.3390/ma18153492/s1, Figure S1: Metals (Pb, Cr and Cu) concentration [mg/Kg] determined in the substrate (in absence of lettuce), the unfertilized control (soil, in the presence of lettuce) and the substrate fertilized with WBF6 (in presence of lettuce). The concentration reported in the plot for Cr concentration was multiplied for 0.2 in order to have a best view. Data are means of five replicates and the error bars represent the standard deviation.

## Abbreviations

The following abbreviations are used in this manuscript:

SRFs	Slow-release fertilizers
WBF	Waste-based fertilizers
ABPs	Animal Byproducts
LD	Soil organic matter
NUE	Nutrient use efficiency
XRF	X-Ray Fluorescence
L.O.I.	Loss on ignition
SSA	Specific surface area
BET	Brunauer–Emmett–Teller
SEM	Scanning Electron Microscopy
CA	Citric acid
AA	Acetic acid
PCU	Polymer-coated urea
HA	Hydroxyapatite
PSB	Phosphate-solubilizing bacteria
