# Supplementary material for: A New High-Efficiency Fertilization System from Waste Materials for Soil Protection: Material Engineering, Chemical-Physical Characterization, Antibacterial and Agronomic Performances
Source: Materials (Basel). 2025 Jul 25;18(15):3492. doi: 10.3390/ma18153492 (PMC12348908; doi:10.3390/ma18153492)
Supplement: Supplementary file 1 [file materials-18-03492-s001.zip › materials-3765052-supplementary.pdf]

Supplementary material

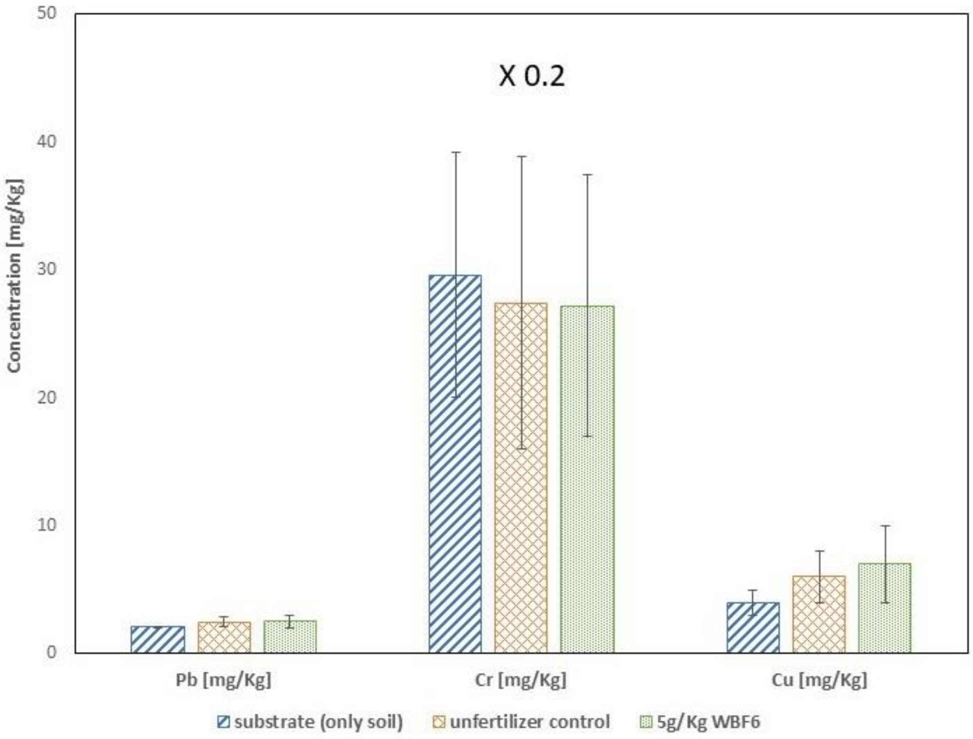

Figure S1. Metals (Pb, Cr and Cu) concentration [mg/Kg] determined in the substrate (in absence of lettuce), the unfertilized control (soil, in the presence of lettuce) and the substrate fertilized with WBF6 (in presence of lettuce). The concentration reported in the plot for Cr concentration was multiplied for 0.2 in order to have a best view. Data are means of five replicates and the error bars represent the standard deviation.
